# Supplementary figures and images for: Immune fingerprinting through repertoire similarity
Source: PLoS Genet. 2021 Jan 4;17(1):e1009301. doi: 10.1371/journal.pgen.1009301 (PMC7808657; doi:10.1371/journal.pgen.1009301)

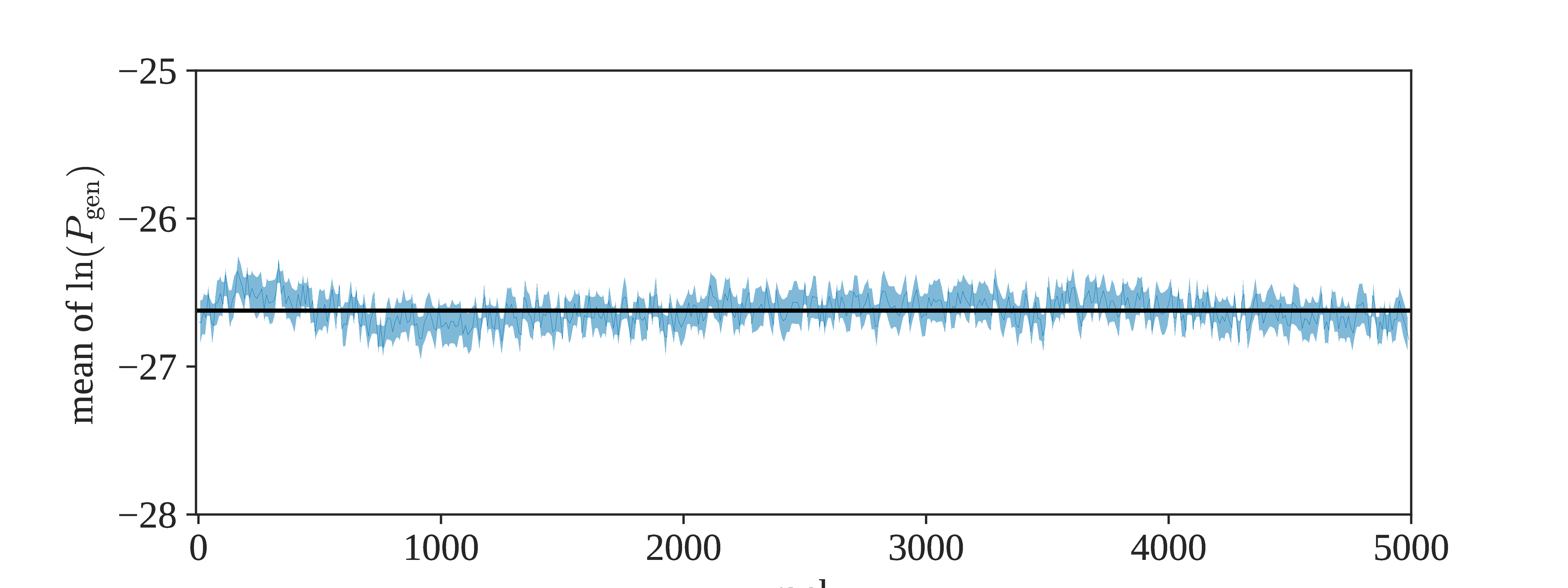

Supplement: S1 Fig — The black line represents the mean of log Pgen for naive clones. The statistic for the top-clones (low rank) is similar to the one for the naive clones. (TIF) [file pgen.1009301.s001.tif]

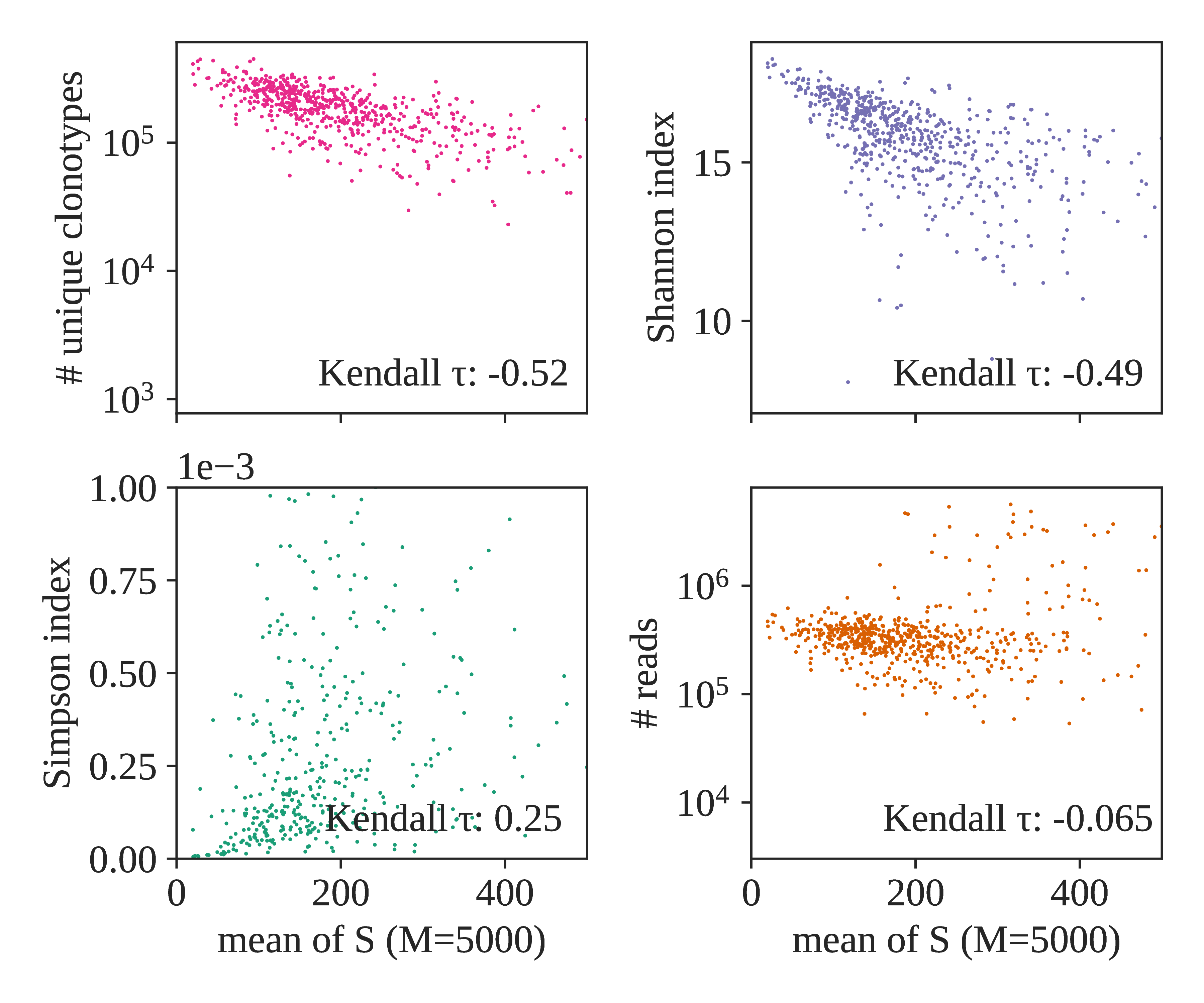

Supplement: S2 Fig — The number of unique sequences found in the dataset (top left), the Shannon index, -∑f^slnf^s (top right), the Simpson index (bottom left), and the total number of reads in each datasets (bottom right). All the diversity measures show a strong correlation with S, but the correlation with the sequencing depth is low. (TIF) [file pgen.1009301.s002.tif]

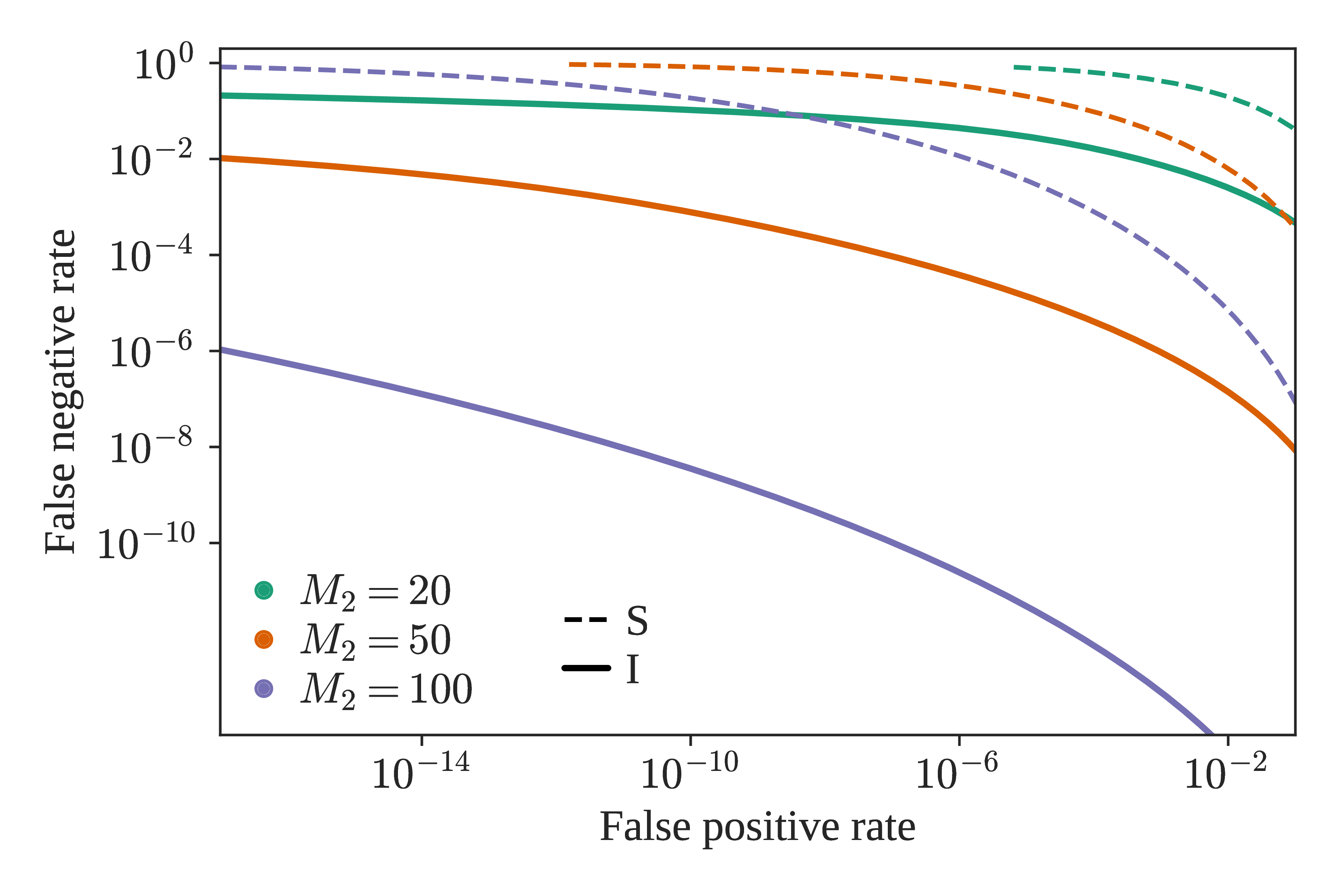

Supplement: S3 Fig — (TIF) [file pgen.1009301.s003.tif]

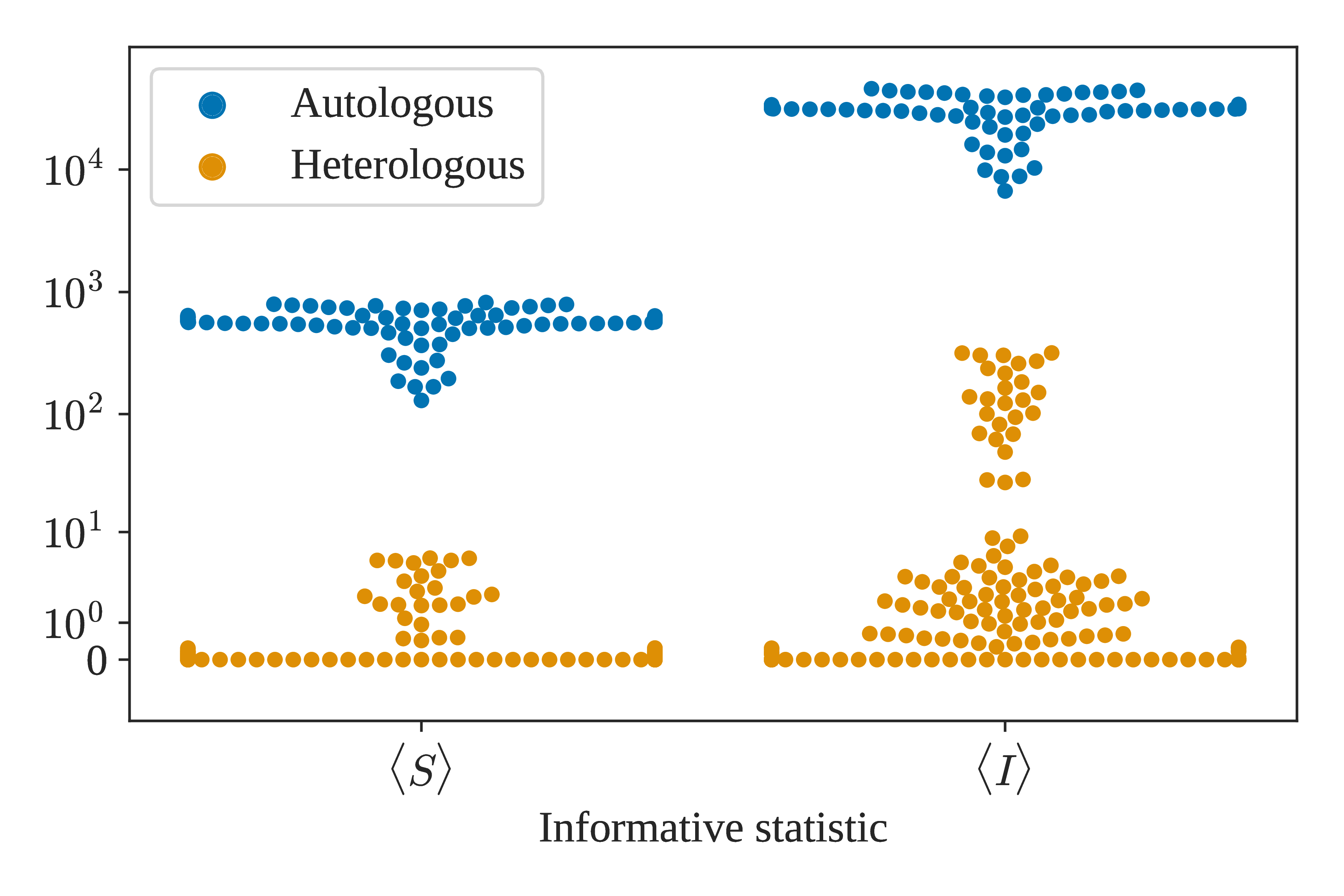

Supplement: S4 Fig — The IGH sequences used are restricted to IgG+ B-cells (selected according to their CH gene). The sequences were obtained from 8 different individuals (6 biological replicates each) in the dataset from [30]. Autologous (blue) and heterologous (yellow) are well separated. (TIF) [file pgen.1009301.s004.tif]

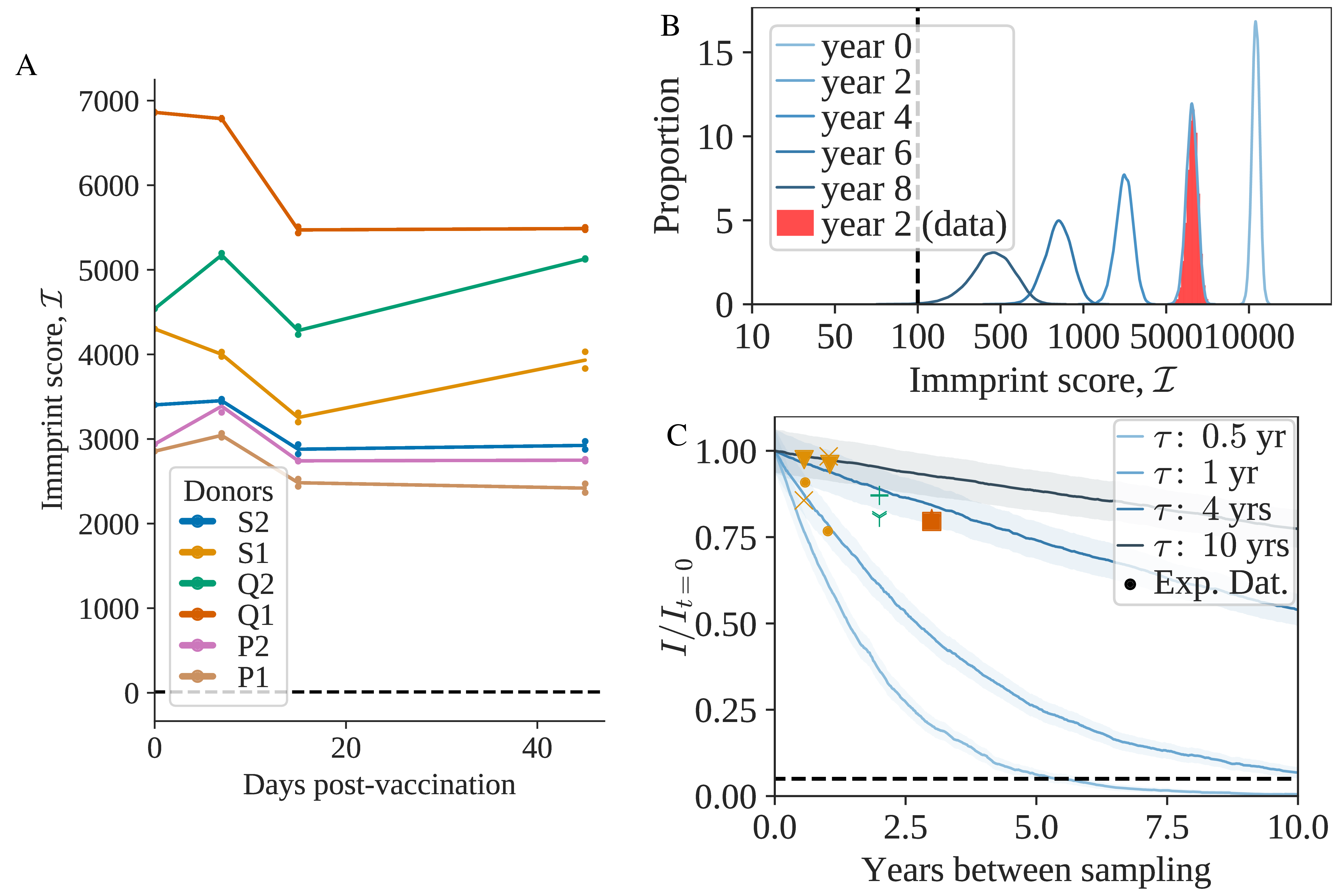

Supplement: S5 Fig — A) Evolution of S (M = 5000) during vaccination, between a sample taken at day 0 (vaccination date) and at a later timepoint. Each color represents a different individual. Each pair timepoint/individual has two biological replicates. The dashed line represents the threshold value. B) Evolution of S between a sample taken at year 0 and a later timepoint. The red histogram corresponds to one of the individuals sampled in [18] and the blue curves show theoretical estimates, fitted to match (τ = 0.66). C) Evolution of the (normalized) mean of S (M = 5000) as a function of time for different values of the turnover rate τ. The dashed line represents the threshold value divided by the smallest value of St=0 (M = 5000) in the data. The data points were obtained from the datasets [35] (yellow), [18] (green) and [22] (orange). Different markers indicate different individuals. (TIF) [file pgen.1009301.s005.tif]

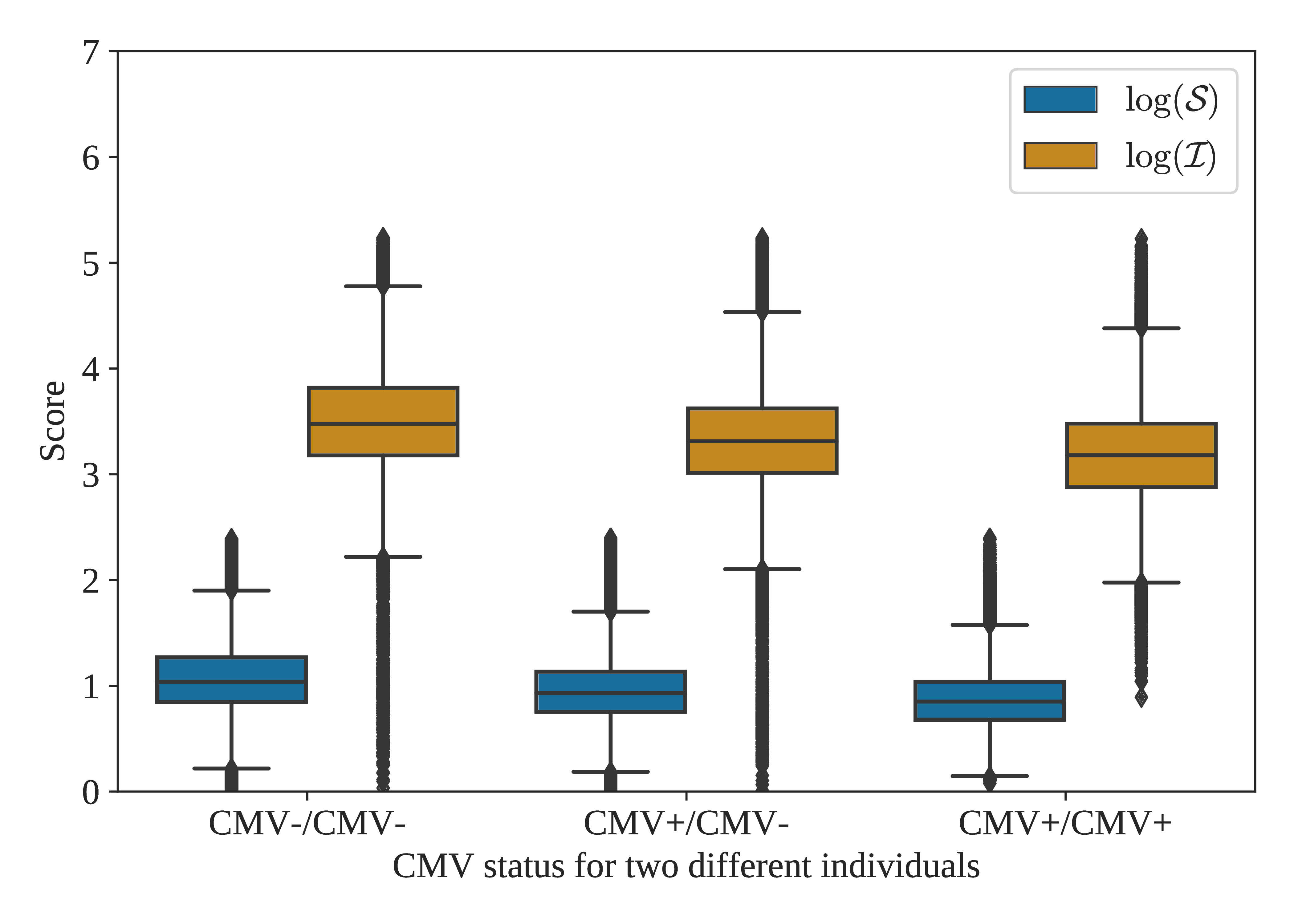

Supplement: S6 Fig — (TIF) [file pgen.1009301.s006.tif]

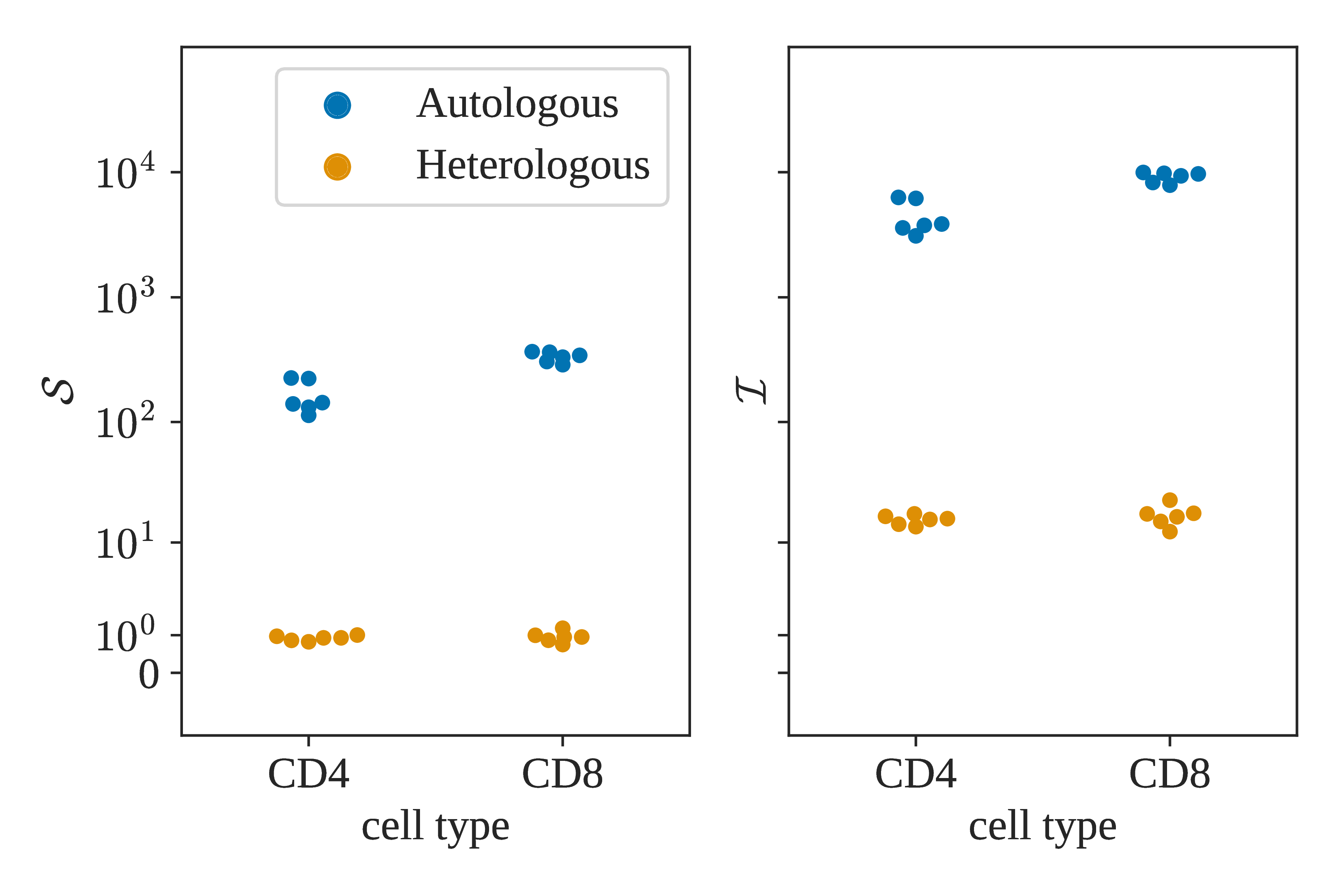

Supplement: S7 Fig — (TIF) [file pgen.1009301.s007.tif]

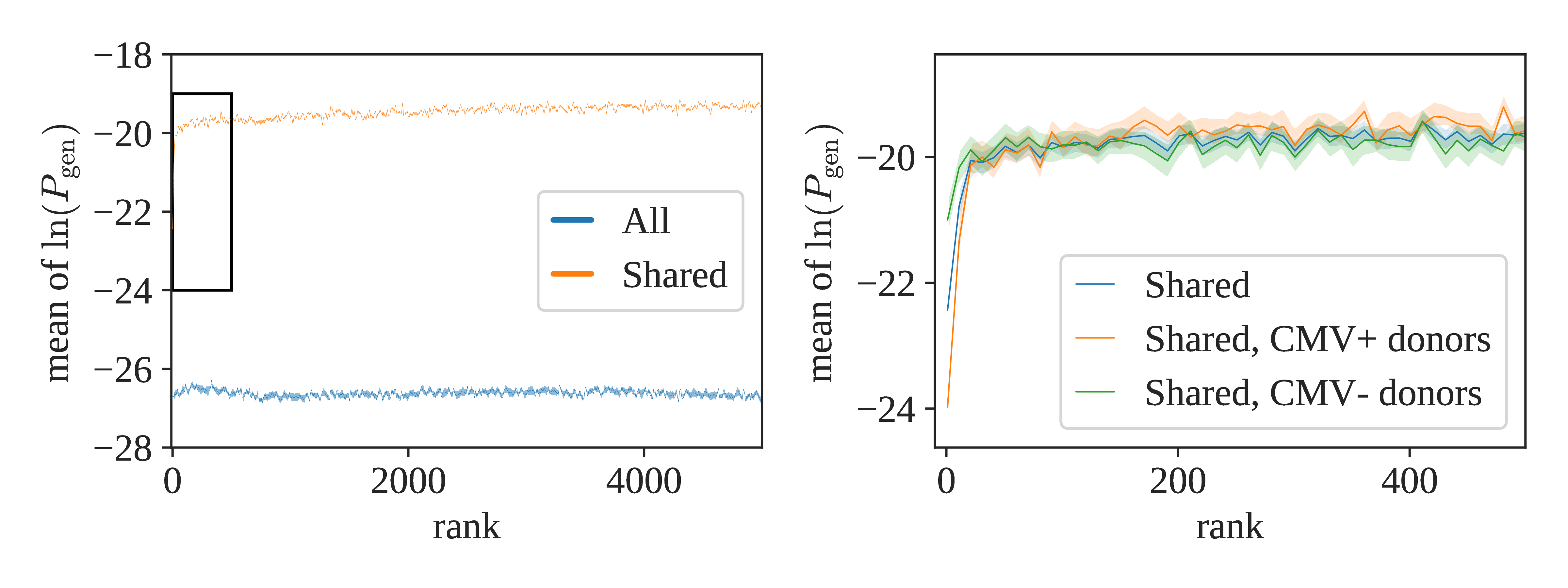

Supplement: S8 Fig — The mean stays flat indicating that the probability of being generated does not generally depend on the clonotype size. There is an exception (black rectangle), shown as a close-up on the right panel. The top twenty clones, when shared between donors, have a smaller probability of being generated than expected by chance. This difference is likely to be driven by convergent selection against common pathogens, since CMV positive donors show a more prononced effect than CMV negative ones. (TIF) [file pgen.1009301.s008.tif]

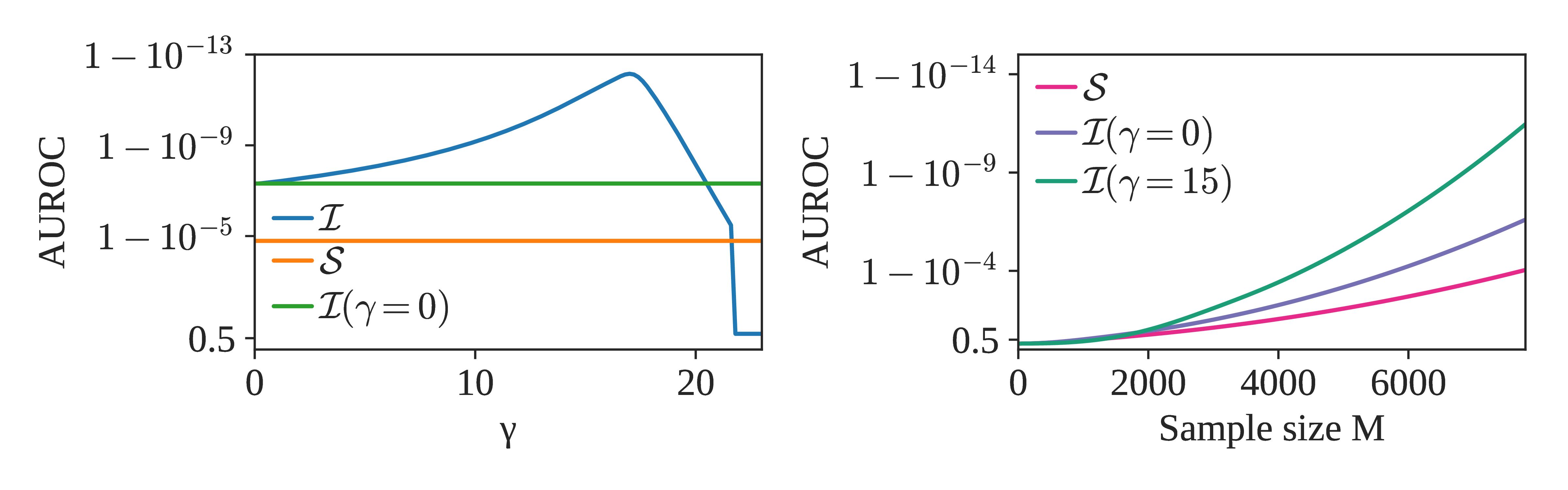

Supplement: S9 Fig — We observe an optimum near γ = 15. Right panel: AUROC as a function of M, for S, I(γ=0), and I(γ=15). (TIF) [file pgen.1009301.s009.tif]

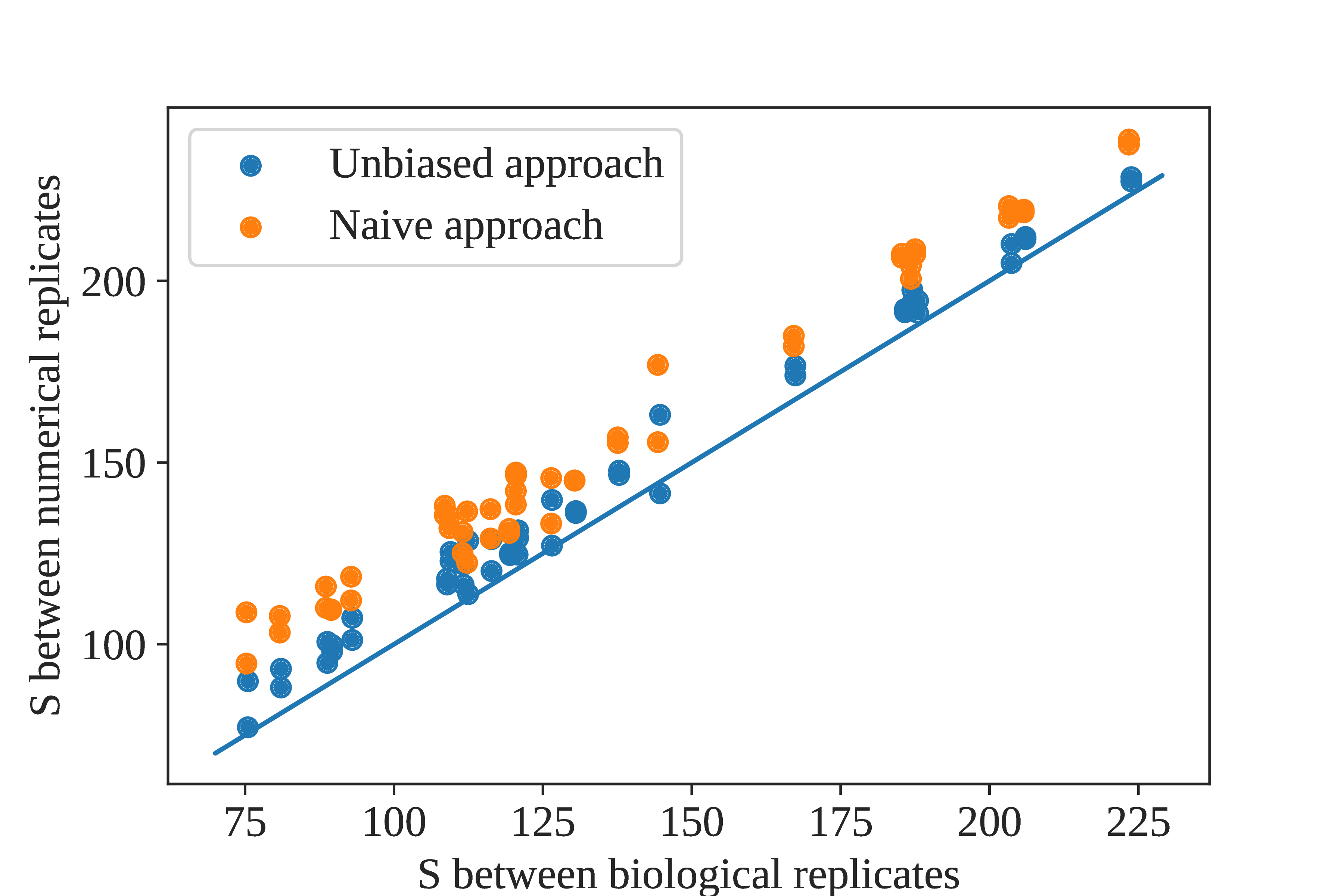

Supplement: S10 Fig — (TIF) [file pgen.1009301.s010.tif]

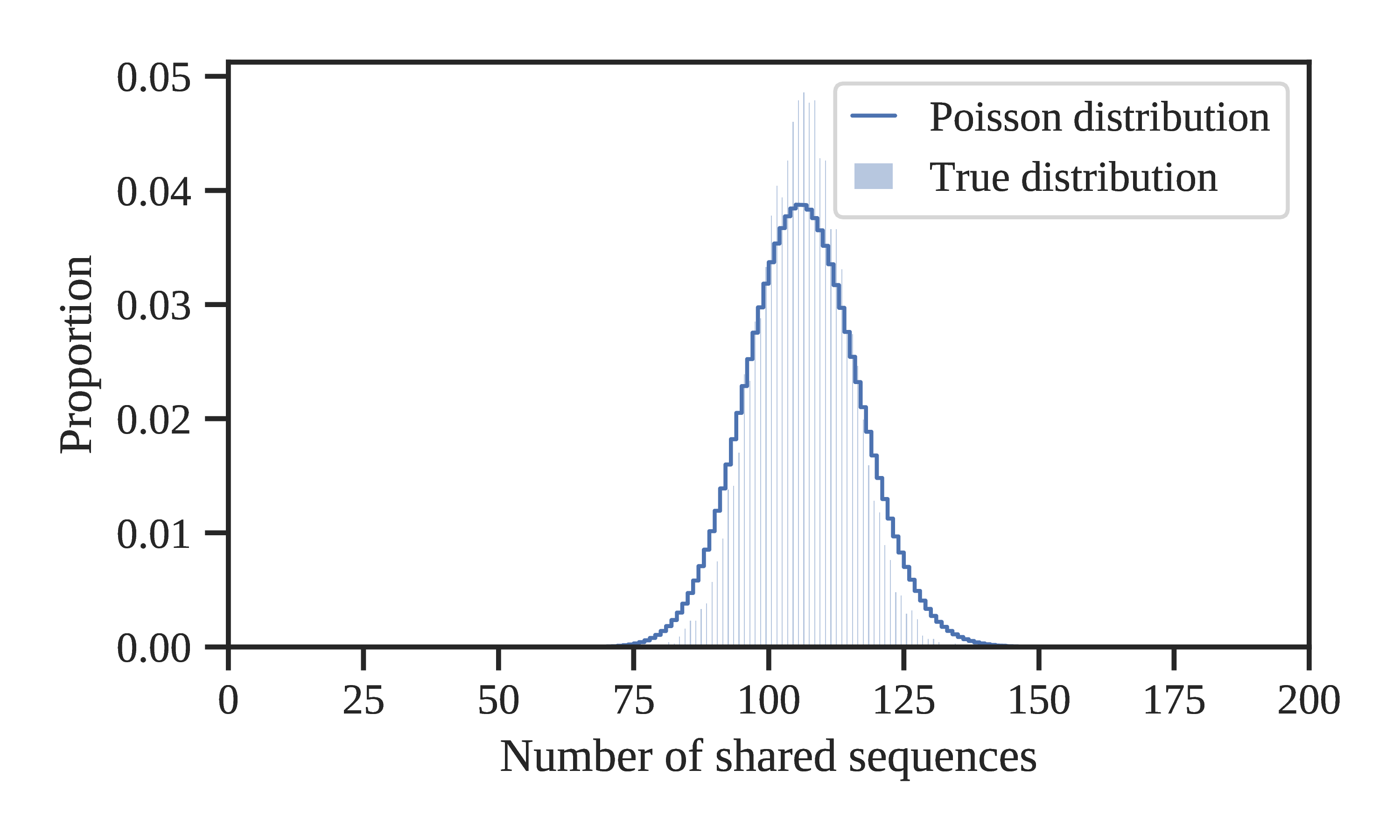

Supplement: S11 Fig — (TIF) [file pgen.1009301.s011.tif]

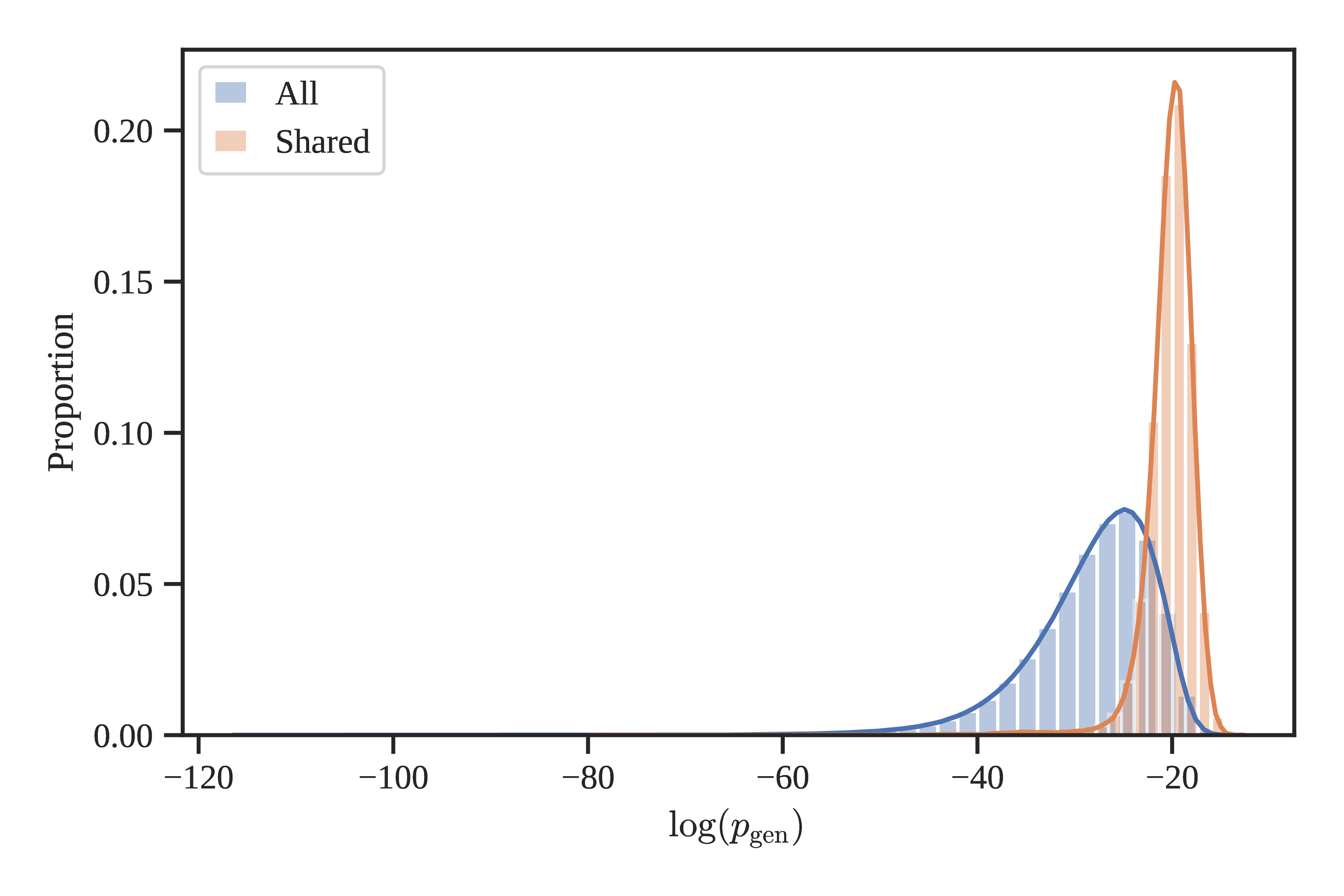

Supplement: S12 Fig — (TIF) [file pgen.1009301.s012.tif]
